# Antibody enhanced HPLC for serotype-specific quantitation of polysaccharides in pneumococcal conjugate vaccine

**DOI:** 10.1038/s41541-022-00584-9

**Published:** 2023-01-23

**Authors:** James Z. Deng, Nathan Kuster, Ashley Drumheller, Mingxiang Lin, Frances Ansbro, Milica Grozdanovic, Rachelle Samuel, Ping Zhuang

**Affiliations:** 1 Vaccine Analytical Research & Development, Analytical Research & Development, MRL, Merck & Co., Inc., Rahway, NJ USA; 2 Analytical External Capabilities, Analytical Research & Development, MRL, Merck & Co., Inc., Rahway, NJ USA; 3 Cell-Based Sciences, Analytical Research & Development, MRL, Merck & Co., Inc., Rahway, NJ USA

**Keywords:** Bacterial infection, Pharmaceutics

## Abstract

Bacterial infection remains as one of the major healthcare issues, despite significant scientific and medical progress in this field. Infection by *Streptococcus Pneumoniae* (*S. Pneumoniae*) can cause pneumonia and other serious infectious diseases, such as bacteremia, sinusitis and meningitis. The pneumococcal capsular polysaccharides (CPS) that constitute the outermost layer of the bacterial cell are the main immunogens and protect the pathogen from host defense mechanisms. Over 90 pneumococcal CPS serotypes have been identified, among which more than 30 can cause invasive pneumococcal diseases that could lead to morbidity and mortality. Multivalent pneumococcal vaccines have been developed to prevent diseases caused by *S. Pneumoniae*. These vaccines employ either purified pneumococcal CPSs or protein conjugates of these CPSs to generate antigen-specific immune responses for patient protection. Serotype-specific quantitation of these polysaccharides (Ps) antigen species are required for vaccine clinical dosage, product release and quality control. Herein, we have developed an antibody-enhanced high-performance liquid chromatography (HPLC) assay for serotype-specific quantitation of the polysaccharide contents in multivalent pneumococcal conjugate vaccines (PCVs). A fluorescence-labeled multiplex assay format has also been developed. This work laid the foundation for a serotype-specific antigen assay format that could play an important role for future vaccine research and development.

## Introduction

Worldwide, diseases caused by *Streptococcus Pneumoniae* are responsible for the major proportion of deaths in children under 5 years of age and adults over 50. Patients can be infected by different serotypes of the bacteria simultaneously or at different times, either continuously or intermittently. By far, more than 90 *S. Pneumoniae* strains have been identified and over 30 of them can cause streptococcus infections^1,2^. *Streptococcus Pneumoniae* are encapsulated gram-positive bacteria. Its immunogenic factors largely come from capsular polysaccharides (CPS) that constitute the bacterial cell wall^3–6^. Each pneumococcal serotype (ST) infects the host with a specific CPS antigen. Each CPS antigen is a unique polysaccharide (Ps) polymer with its own repeating unit (RU) structure (ST4 example in Supplementary Fig. 1). Conjugation of polysaccharide to a carrier protein, such as CRM197 help to recruit helper T cells, and offer long-lasting and more robust T-cell-dependent immune responses^7–12^. Although two powerful pneumococcal conjugate vaccines (PCVs), PREVNAR 20 and VAXNEUVANC have been approved by FDA recently, there are still great needs for novel pneumococcal vaccines that can offer more robust immune responses and greater serotype coverages^13–16^. Analytical methods that monitor critical quality attributes (CQA) in such complex multi-valent vaccine product with increasing serotype repertoire often encounter challenges, but are critical for the success of vaccine development^17–19^.

**Fig. 1:**
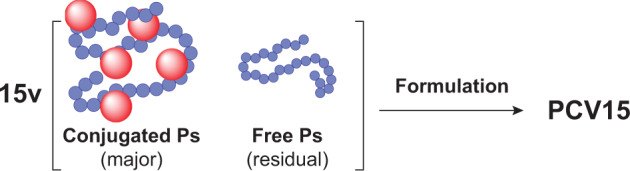
PCV15 vaccine product. CRM197 protein-conjugated polysaccharide (Ps) is the major antigenic form, and that unconjugated free Ps is a minor form. In PCV15 vaccine, 15 types of conjugate and free polysaccharides are formulated together.

Our 15-valent pneumococcal conjugate vaccine (PCV15) consists of pneumococcal CPS serotypes 1, 3, 4, 5, 6A, 6B, 7F, 9V, 14, 18C, 19A, 19F, 22F, 23F, and 33F. Each CPS is conjugated to the carrier protein CRM197 individually, resulting a monovalent conjugate polysaccharide (conjugated Ps). Residual amount of unconjugated polysaccharide species (free Ps or free polysaccharide) also coexists with conjugated Ps in the drug substance (Fig. 1). Both conjugated Ps and free Ps can elicit serotype-specific immune responses once dosed to the patient, with the conjugate Ps being the more desired antigen form that can elicit long-lasting T-cell-dependent immune responses.

To assess the potency and quality of the vaccine, a serotype-specific polysaccharide quantitation assay will need to be implemented in vaccine development and manufacturing. Conventionally, such serotype-specific antigen assays were performed on the immunoassay platforms; among them, the sandwich enzyme-linked immunosorbent assay (ELISA) is the preferred format for this assay^20,21^. Although immunoassays, such as ELISA have been reported to quantify antigen content in several vaccines^22–24^, there is only a light scattering (LS)-based nephelometry method reported for pneumococcal vaccines^25^. Due to the lack of precedent reports for a reliable antigen-specific pneumococcal immunoassay and the need for such assay for our PCV development, we sought to develop an innovative assay platform for serotype-specific polysaccharide quantitation. For the last few decades, high-performance liquid chromatography (HPLC) has been demonstrated as a highly robust and automatic assay platform for vaccine analysis^26,27^. HPLC methods have also been utilized to report the concentration of a single polysaccharide or total polysaccharides in a sample^28–30^. However, HPLC methods have not been established for serotype-specific analysis for a sample containing multivalent antigens. The polysaccharides in the PCV are large molecular weight (Mw ~100–500 kDa), polydisperse and flexible biopolymers with overlapped monosaccharide components in their repeating unit (RU) structures^31,32^. Therefore, direct chromatography separation and detection were not able to deconvolute each serotype in a multivalent vaccine.

**Fig. 2:**
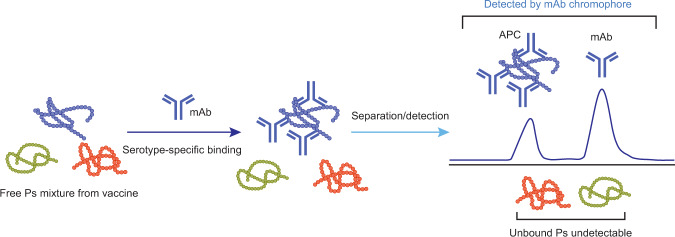
Antibody-enhanced chromatography for serotype-specific polysaccharide quantitation. A serotype-specific antibody would only bind to its corresponding polysaccharide serotype in the vaccine. The resulting type-specific antibody–polysaccharide complex is separated from excess antibody and used for polysaccharide quantitation.

We proposed that we could establish a novel antibody-enhanced chromatography assay for serotype-specific antigen quantitation by leveraging the specificity of each anti-serotype (anti-ST) antibody (Ab). An anti-ST Ab binds to only its corresponding polysaccharide serotype without cross-reactivity to untargeted serotypes. By complexing of an anti-ST Ab and multi-polysaccharide serotypes together in an antibody–antigen binding reaction, a serotype-specific antibody–polysaccharide complex (APC) would form in solution. The serotype-specific APC can therefore serve as a surrogate to analyze the target polysaccharide serotype. Once the APC is separated from the excess unbound antibody, the APC peak area on the chromatogram (or electropherogram) can be used to quantify the antibody-targeted polysaccharide serotype. The unbound serotypes (untargeted) do not need to be separated from either APC or Ab, since they do not have the chromophore (or fluorophore) used for detection. Only species that hold antibody (either APC or antibody itself) are visible as peaks on the chromatogram (Fig. 2).

**Fig. 3:**
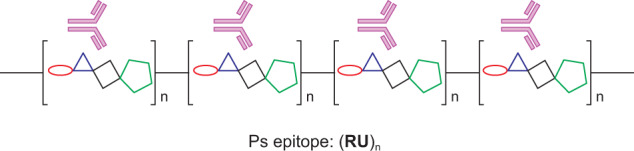
Anti-ST antibody binding to epitopes on a polysaccharide. Each antibody-binding epitope is formed by a number (*n* = 3–9) of polysaccharide repeating unit (RU). Each RU is formed by a few individual monosaccharides represented by different shapes in the bracket. When all epitopes are saturated by antibody binding, the amount of antibody bound on the polysaccharide can reflect the polysaccharide amount.

According to structural studies^33–37^, an anti-serotype antibody binds to a defined polysaccharide structural motif (epitope). The polysaccharide epitope is formed by a defined number of repeating units (RUs). One polysaccharide molecule has multiple antibody-binding epitopes, with each epitope consisting of a defined number of RUs (Fig. 3). When the epitopes on the polysaccharide are saturated by the antibody binding, the number of antibodies on the resulting APC will represent the number of RUs on the polysaccharide, which can be converted to molar concentration of RU (mM), or weight concentration of the polysaccharide (µg/mL) based on the known molar mass (Mw or molecular weight) of the specific RU. Guided by this structural information, we established a serotype-specific assay that quantifies the free polysaccharide content in our PCV15 vaccine.

**Fig. 4:**
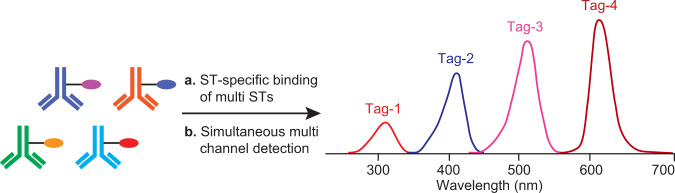
Analysis of multi-valent PCV on the multiplex chromatography assay platform with fluorescent (FLR) tag-labeled antibodies. Each of the four antibodies was labeled with a distinctive FLR tag. On HPLC, the detection was set on the specific *Ex/Em* wavelength unique to the tag. The CRM protein on the conjugate was not labeled.

Conjugated polysaccharide in a PCV bear a carrier protein that could interfere with antibody-assisted polysaccharide quantitation in its original format. Both antibody and CRM197 have signal at *Ex/Em* 280 nm/352 nm used for fluorescence detection (or UV 280 nm absorption), since they both are proteins. This conundrum can be resolved by labeling the anti-serotype antibody with a fluorescent tag (FLR tag) and switching the detection channel to the wavelength that is unique to the fluorescent tag, e.g., *Ex/Em* 433 nm/541 nm, at which unlabeled CRM197 proteins on the conjugate do not have a detectable signal. Hence, the FLR tag-labeled antibody chromatography can be used to quantify the protein-conjugated polysaccharide. Furthermore, simultaneous detections at multiple wavelengths can be easily set up on modern HPLC instruments. When different anti-serotype antibodies are labeled with several distinctive FLR tags, the signal from these preselected FLR tags can be detected and resolved on multidetection channels on a HPLC. Therefore, multiple conjugated polysaccharide serotypes in a complex multivalent vaccine can be analyzed in a single HPLC run (Fig. 4), and a multiplex serotype-specific antigen assay platform was developed.

## Results

### Development of serotype-specific HPLC assay

**Fig. 5:**
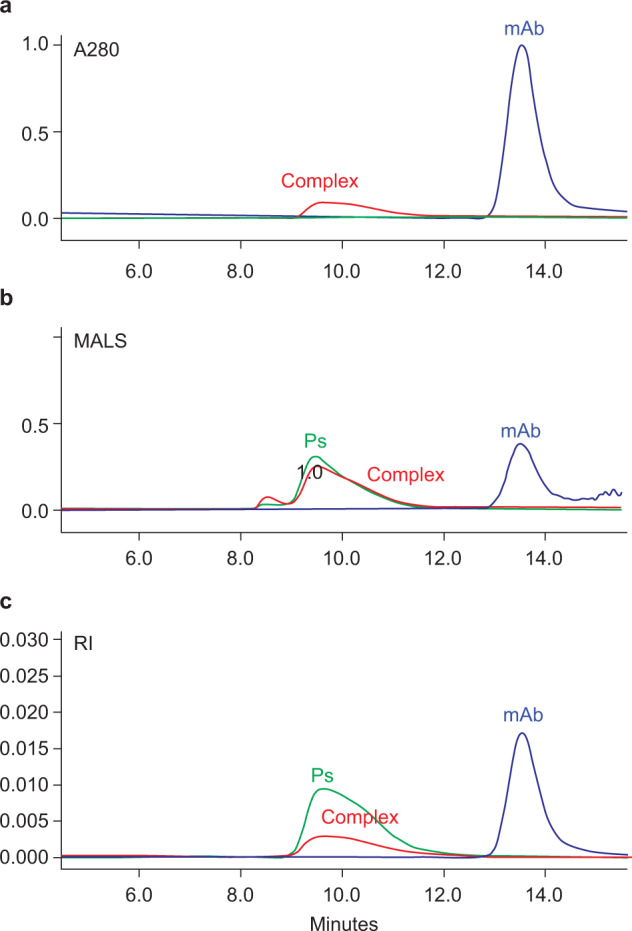
Detection and analysis of antibody–polysaccharide complex (APC) on SEC-UV-MALS-RI. **a** APC peak (red) detected by UV 280 nm (A280). **b** APC peak (red) and unbound polysaccharide peak (green) detected by multi-angle light scattering (MALS); **c** APC (red) and unbound polysaccharide (green) peaks detected by refractive index (RI) detector.

Since we had previous successful size-exclusion chromatography (SEC) methods for the vaccine, free Ps, and protein-conjugated Ps characterization and quantitation^38–40^, we decided to start our assay development on the same platform. An anti-ST1 mAb was mixed with pneumococcal serotype 1 (ST1) Ps standard and injected on the SEC-UV-MALS-RI system (“Methods”). A higher Mw peak that was separated from the mAb was observed on all three detection channels, whereas the un-complexed Ps could not be detected on UV A280. This substantiated the formation of antibody–polysaccharide complex (APC), which was detected with the chromophore acquired from the mAb at A280 (Fig. 5 and Table 1).

**Table 1:** SEC-UV-MALS-RI analysis of complexes formed by anti-ST1 mAb and ST1 Ps.

mAb-ST1 complex (APC)	mAb/Ps ratio (µg/µg) in binding reaction	Mw (kDa)
Anti-ST1 mAb STD	NA	204
ST1 Ps STD	NA	300
APC Complex-1	0.25	571
APC Complex-2	0.5	700
APC Complex-3	1.0	874

**Fig. 6:**
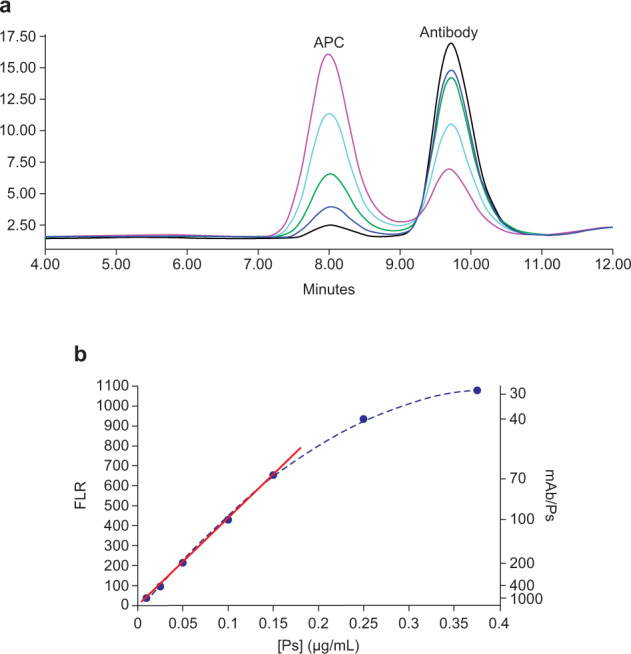
Antibody titration of ST4 polysaccharide. **a** Chromatogram overlay of antibody–polysaccharide complex (APC) formed under five different Ps concentrations. APC is eluted out at ~8 min and is separated from unbound antibody (eluted at ~9.8 min) (**b**). A 7-point polysaccharide antibody titration curve. With same antibody concentration at each point, a linear response was observed when polysaccharide concentration ([Ps]) is ≤0.15 µg/mL. The linear line started to bend when polysaccharide concentration increased to above 0.15 µg/mL, where antibody to polysaccharide ratio dropped below what is required to saturate all binding epitopes on the polysaccharide.

We then moved to probe the assay linearity range of one serotype with fluorescence (FLR) detection, so we could later expand the assay to remaining serotypes. Under binding conditions with excess mAb *vs* serotype 4 (ST4) polysaccharide (Ps), the ST4 antibody–polysaccharide complexes (APCs) were observed on the SEC chromatogram at all Ps concentration levels (Fig. 6a), and a linear FLR response curve was established from the polysaccharide (Ps) concentration range of 0.01 to 0.15 µg/mL (Supplementary Table 1). As Ps concentration increased above 0.15 µg/mL, the FLR response curve started to flatten, indicating that the multi-epitopes on a polysaccharide molecular structure were no longer saturated by mAb binding at these concentrations (Fig. 6b). Furthermore, the binding reaction was probed at different time points (0.5–5 h), to establish a robust assay window where results would not be impacted by the binding kinetics. Assay results are very consistent between one to five hours of binding time (Supplementary Table 2 and Supplementary Fig. 2).

### Initial analytical performance assessment

After established proof-of-concept on ST4, the assay was expanded to the remaining PCV15 serotypes for free polysaccharide (free Ps) quantitation. Initial assessments of assay performance parameters required by FDA and ICH for method validation^41,42^ were performed in the following sections. These parameters include assay specificity, linearity, range, precision and accuracy as discussed in the following paragraphs in this section.

The serotype (ST) knockout (KO) sample (“Methods”) for each of the PCV15 serotypes was prepared to demonstrate assay specificity. Antibody–polysaccharide complex (APC) was only observed in the binding reaction of an anti-ST monoclonal antibody (mAb) with the Ps of the same serotype. There is no APC observed in the reaction between an anti-ST mAb and the corresponding serotype KO sample. (Supplementary Fig. 3). This suggests that APC arises from the serotype-specific binding between the anti-ST mAb and Ps of the same serotype.

An anti-ST mAb was bound to the corresponding serotype standard at concentrations from low to high to generate a 5-point standard curve for each serotype. The standard curve was demonstrated by APC fluorescence signal peak areas *vs* the polysaccharide concentrations in these binding reactions. Excellent linearity (*R*^2^ > 0.99) has been observed for fourteen of the 15 serotypes, good linearity (*R*^2^ > 0.98) has been observed for ST33F. The standard curve range is from 0.01 to 0.15 µg/mL for most serotypes, or lower for some serotypes (Table 2 and Supplementary Fig. 4). This demonstrates that the assay is capable of serotype-specific quantitation of polysaccharide at sub-µg/mL levels.

**Table 2:** Standard curve linearity and range for 15 serotypes of pneumococcal Ps.

PCV15 serotype	1	3	4	5	6A	6B	7F	9V	14	18C	19A	19F	22F	23F	33F
Avg *R*^2^ (*n* = 3)	0.9998	0.9998	0.9999	0.9997	0.9982	0.9995	0.9952	0.9999	0.9997	0.9998	0.9999	0.9997	0.9992	0.9998	0.9809
Range (µg/mL)	0.005–0.050	0.010–0.100	0.010–0.150	0.010–0.150	0.010–0.150	0.005–0.050	0.010–0.150	0.010–0.150	0.010–0.150	0.010–0.150	0.010–0.150	0.010–0.150	0.005–0.100	0.010–0.150	0.010–0.150

Precision for this assay was evaluated by three experiments on two instrument systems. Before binding to a mAb, the PCV15 vaccines were prepared by an affinity capture step to remove CRM197 conjugate species. The resulting vaccine sample containing only free polysaccharide was bound to each of the fifteen anti-ST mAb individually in a binding reaction that mimicked the polysaccharide standard. The vaccine binding reactions were analyzed on SEC HPLC, and the APC peak for each ST was compared with the corresponding standard curve to calculate the free Ps in the vaccine (Supplementary Fig. 5). The precision for all 15 serotypes is summarized in Table 3. The serotype-specific chromatography assay has precisions better than 13.3% CV(coefficient of variation) for all serotypes.

**Table 3:** Precision for free Ps quantitation.

	Free [Ps] (µg/mL) in PCV15														
Serotype	1	3	4	5	6A	6B	7F	9V	14	18C	19A	19F	22F	23F	33F
Exp-1	0.323	0.408	0.109	0.425	0.126	0.346	0.140	0.140	0.149	0.080	0.784	0.200	0.143	0.106	0.272
Exp-2	0.325	0.371	0.104	0.428	0.140	0.328	0.108	0.138	0.143	0.083	0.764	0.207	0.148	0.105	0.263
Exp-3	0.327	0.377	0.102	0.439	0.120	0.270	0.119	0.136	0.130	0.083	0.731	0.184	0.147	0.126	0.333
Avg	0.325	0.385	0.105	0.431	0.129	0.315	0.122	0.138	0.141	0.082	0.760	0.197	0.146	0.112	0.289
%CV	0.7	5.2	3.2	1.8	7.8	12.5	13.3	1.7	6.8	2.0	3.5	5.9	2.0	10.7	13.3

Assay accuracy was evaluated with six representative serotypes. The standard curve has a range of 0.01–0.15 µg/mL for these serotypes. The middle of the highest standard concentration (0.075 µg/mL) is considered as the nominal assay concentration, and one-fifth of the nominal concentration (0.015 µg/mL) as lower limit of quantitation (LLOQ). The assay accuracy was assessed by the spike recoveries at the nominal concentration and at LLOQ. For all tested STs, excellent spike recoveries (97–103%) were observed for the spikes at nominal concentration. Spike recoveries at LLOQ were also acceptable^42^ at 83–119% (Table 4 and Supplementary Table 3). These demonstrate good assay accuracy. The free polysaccharide from the antibody chromatography assay are generally in agreement with results from our in-house sandwich ELISA assay and are in line with free Ps input expected from the vaccine formulation (Supplementary Table 4).

**Table 4:** Accuracy assessed by sipke recovery.

Serotype	ST4	ST5	ST6A	ST9V	ST14	ST19A	Overall
% Spike recovery at LLOQ	83	92	95	119	115	101	83–119
%Spike recovery at nominal	97	101	97	102	103	98	97–103

### Multiplex serotype-specific chromatographic assay for conjugate quantitation

With the serotype-specific free Ps assay in hand, we sought to expand the assay to include conjugated polysaccharides. The challenge for a conjugate assay derives from the fact that protein signal detected from a serotype-specific APC complex not only represents mAbs binding to polysaccharides, but also CRM197 carrier proteins already on the conjugate. To mask out the background signal from the carrier protein, Alexa Fluor (AF) fluorescent (FLR) tag-labeled anti-ST mAb was generated and the fluorescence-tagged mAb was used for binding. The resulting fluorescence-labeled APC was detected by the FLR tag signal in a channel distinctive from that used for native protein detection (*Ex/Em* 280/352 nm). This FLR-labeled antibody chromatography assay can also be adapted on a multiplex format, since HPLC detectors have the ability to do multi-channel FLR or UV detection simultaneously. With this in mind, we labeled four anti-ST mAbs, each with a unique FLR tag (anti- ST4-AF350, ST5-AF430, ST6A-AF555, and ST14-AF633, “Methods”) (Supplementary Fig. 6). Four distinctive fluorescence channels were set in a single HPLC method, each is corresponding to one of the FLR tags on a mAb. In this way, one serotype can only be detected on the desired FLR channel, with no signal observed on the other three channels (Supplementary Fig. 7). Coupled with antibody’s binding specificity, a multiplex serotype-specific assay for PCV total Ps (conjugated + free Ps) has been established. It is worth to note that this multiplex assay can be applied to both conjugated and unconjugated polysaccharides.

**Fig. 7:**
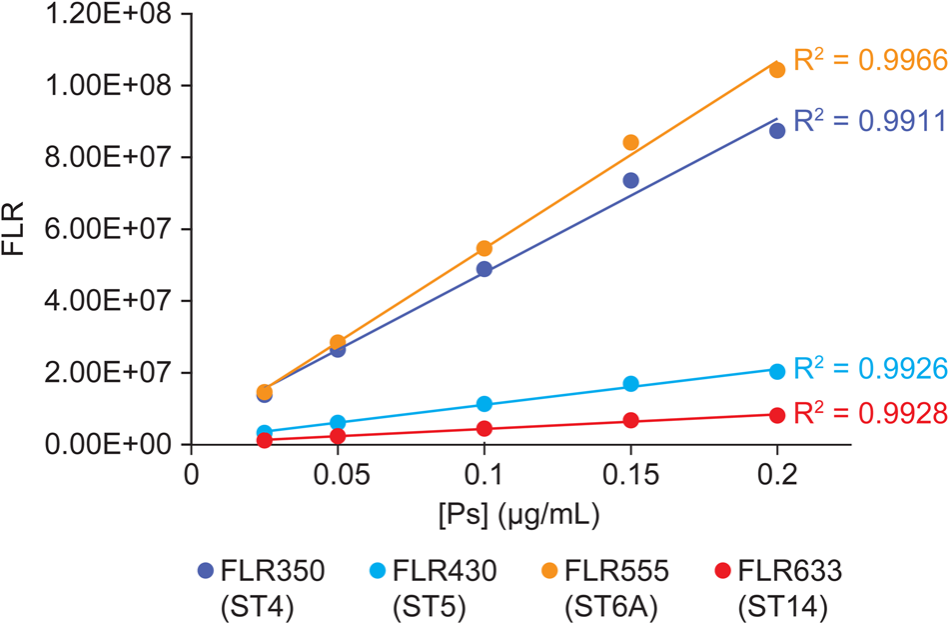
Overlay of standard curves from multiplexing fluorescence (FLR)-tagged mAbs with a multivalent PCV standard. The four standard curves were generated in a single HPLC run by simultaneous detections on four fluorescence channels.

A PCV vaccine standard with conjugated ST4, ST5, ST6A, and ST14 were used to generate four standard curves in a single HPLC run. All four conjugate serotypes showed good linearity in the respective standard curve (*R*^2^ > 0.99) (Fig. 7 and Supplementary Table 5). A 24-valent conjugate vaccine (PCV24)^43^ with a target formulation total Ps (conjugate Ps + free Ps) concentration of 8 µg/mL for each serotype was analyzed in a single HPLC run. The measured Ps concentrations are within ±11% of the target potency value (Table 5 and Supplementary Table 6) for all four tested serotypes (ST4, ST5, ST6A, and ST14), complying with the normal acceptance criteria of ±30% of the label claim for polysaccharide conjugated vaccines^23,25^. The 4-in-1 multiplex assay format reduces the HPLC runs needed to fully quantify all serotypes in the PCV24 from 24 to 6.

**Table 5:** Analysis of four PCV serotypes in PCV24 vaccine by multiplex antibody chromatography.

PCV24 total Ps	ST4	ST5	ST6A	ST14
FLR detection channel	FLR350	FLR430	FLR555	FLR633
Peak area (µV*sec)	52,581,520	11,054,715	49,747,133	4,163,950
Binding Rx [Ps] (µg/mL)	0.111	0.100	0.091	0.095
Sample dilution	80	80	80	80
PCV24 total [Ps] (µg/mL)	8.9	8.0	7.3	7.6
% difference from target	11.0	0.2	9.3	5.1

## Discussion

Pneumococcal disease is a major cause that leads to serious medical conditions. Vaccine development for this disease can be greatly benefited from a simple and robust serotype-specific antigen assay for vaccine products. Previous reports of such assay for pneumococcal conjugate vaccines are scarce and lack of recent advance. The only literature-reported method is a nephelometry method. The nephelometry method uses light scattering to detect the antibody–polysaccharide complex formed in the solution. The nephelometry observed 82–119% variation from specification. The linearity range for this method is rather narrow due to the general non-linear nature of direct light scattering response to concentrations. Also, since the assay is performed in a direct batch flow-through mode without separation of or washing off sample matrix, sample formulation matrix could potentially impact the quality of the results. For certain conjugate sample, treatment by trypsin may be required to achieve optimal result in nephelometry^25^. In the antibody-assisted HPLC assay, antibody–polysaccharide complex is separated from sample matrix on chromatography. Therefore, matrix interference can be minimized. The only sample preparation procedure is the dilution with sample or binding buffer before analysis. Generally, a 10-fold to 15-fold linearity range can be easily established on an antibody HPLC assay.

Presumably, another preferred assay platform for such serotype-specific quantitation would be the sandwich ELISA assay, although there is no literature report about its application on a pneumococcal vaccine. For each serotype, sandwich ELISA assay platform would require three antibodies: a capture antibody, a detection antibody and an enzyme-linked antibody for the chemiluminescence signal. Several concentration points would be required for generating a titration curve in ELISA analysis. ELISA sample and plate preparations involve relatively manual and lengthy processes. In antibody-assisted HPLC, sample preparation and antibody binding are relatively simple and straightforward. The HPLC analysis is automatic and can be easily transferred between labs without much variation. Antibody-assisted HPLC only requires one antibody for each serotype in comparison with three for the sandwich ELISA. This will reduce some burden on critical reagent generation and management. Even though antibody-assisted HPLC is benefit from signal enhancement from binding multiple antibodies to one polysaccharide, generally it is still less sensitive than an ELISA assay. ELISA could analyze samples at ng/mL concentration, whereas antibody HPLC worked best at ~0.005 to 0.20 µg/mL range in our experience. All assays discussed above could share the same control strategies for reference standards and antipolysaccharide antibody reagents.

Good linearity (generally *R*^2^ ≥ 0.99) has been achieved for the antibody-enhanced HPLC assay. For residual free polysaccharide analysis in the PCV15 vaccine, we have reported precision of %CV from 0.7% to 13.3% across all 15 serotypes. In our matrix based approach to assess assay accuracy, a 97–103% recovery was obtained for samples tested at nominal concentration, with six different serotypes were analyzed. The assay performance meets the regulatory requirements for initial validation assessment of both chromatographic assays (CCs) and ligand binding assays (LBAs)^42^. Since each free polysaccharide serotype existed in different concentrations, these indicated that analytes with varied concentrations performed similarly well in the antibody HPLC assay. If a formal method validation with more sample batches and data points is successful in the future, the assay could be used for commercial product release or quality control. We also established proof-of-concept for using fluorescent tag-labeled antibodies to quantify polysaccharide conjugates on a multiplex HPLC assay platform. A 5–11% variation from product specification was observed for the four tested serotypes. This is well below regulatory criteria of ±30% of specification, for a vaccine content/potency release assay^23,25^. A fourfold improvement on assay throughput would greatly reduce resource needed for analysis of a multi-valent vaccine containing 20 plus serotypes. Further development work is needed to assess the full spectrum of the assay performance on this novel assay format. An antibody-assisted HPLC assay can be easily developed to analyze other polysaccharide and vaccine products, when quality of antibody is reliable and under good control.

Although the antibody HPLC assay was only established on the size-exclusion chromatography (SEC) platform in this report, conceivably it can also be established with other chromatographic separation methods, such as ion-exchange (IEX) or hydrophobic interaction chromatography (HIC) or with electrophoresis separation methods, such as capillary zone electrophoresis (CZE) or capillary isoelectric focusing (cIEF). Furthermore, the antibody-enhanced chromatography assay leveraged on the structure features of antibody binding to a multi-epitope antigen (polysaccharide). This structure-guided assay development approach can be applied and explored for future assay developments. After the assay is established, in turn it can be used to study antibody–polysaccharide binding affinity and kinetics, characterize the antibody–polysaccharide complex and assess quantity and quality of serum antibody obtained from clinical trial subjects or patients.

In this study, a novel antibody-enhanced chromatography assay for serotype-specific quantitation of polysaccharide was developed for a pneumococcal conjugate vaccine. This represents the first time report of a serotype-specific antigen chromatography assay for a multi-valent vaccine. Good assay performance (specificity, linearity, precision and accuracy) has been demonstrated for the quantitation of free polysaccharides in the vaccine. Proof-of-concept was demonstrated on a multiplex HPLC assay platform, when fluorescent tag-labeled antibodies were introduced. This multiplex platform is capable of quantifying both protein-conjugated and unconjugated polysaccharides in a multi-valent vaccine. This simple and automatic assay platform should offer great impact and broad applications in the research and development of vaccines and biologics.

## Methods

### Reagents and materials

Bis-Tris-HCl 1 M solution was purchased from Rigaku Reagents, Inc. (Seattle, WA). Sodium chloride 5 M solution was bought from Promega Corporation (Madison, WI). Pierce™ Protein A/G Magnetic Beads, Alexa Fluor™ NHS Esters and Zeba Spin desalting columns were purchased from Thermo Fisher. PBS buffer was purchased from Gibco. Antibody-binding buffer was made by our in-house reagent group.

### MAbs and FLR tag-labeled mAbs

Pneumococcal anti-ST mAbs and anti-CRM197 mAbs were generated and provided to authors by our in-house antibody generation and critical reagent teams^44,45^. All mAbs were generated in-house for this research. All mAb clones were generated from our internal research teams, except clones for anti-serotypes 1 and 19F mAbs. Anti-serotypes 1 and 19F mAbs were licensed from the University of Alabama at Birmingham (UAB). The mAb species and source were listed in Supplementary Table 7. Each of the anti-polysaccharide mAb was diluted from the stock mAb solution as in Supplementary Table 8, before added into a binding reaction.

FLR tag-labeled anti-ST mAbs were prepared by conjugation to corresponding Alexa Fluor™ NHS esters as exemplified by labeling of anti-ST4 with Alexa Fluor™ 350 (AF350) below.

An anti-ST4 mAb was incubated with excess equivalents of Alexa Fluor™ 350 NHS ester PBS buffer for three hours at ambient temperature. The reaction was purified by desalting through a Zeba Spin desalting column. AF350-labeled anti-ST4 (anti-ST4-AF350) mAb was collected from the column. In the same fashion, anti-ST5, anti-ST6A and anti-ST14 mAbs were prepared as anti-ST5-AF430, anti-ST6A-AF555 and anti-ST14-AF633, respectively (with Alexa Fluor™ 430, Alexa Fluor™ 555 and Alexa Fluor™ 633).

The four FLR-labeled anti-ST mAbs are mixed together as a cocktail (FLR-mAbs) with 0.2 mg/mL of each mAb and used to multiplex with a multivalent PCV vaccine.

### PCV vaccine product and PCV conjugate and free polysaccharide standards

PCV-free polysaccharide standards, conjugated polysaccharide standards, and research batches of PCV vaccine were generated and provided to authors by our vaccine process and formulation groups^46^.

Serotype-specific knockout (KO) polysaccharide (Ps) samples are prepared by authors by mixing fourteen out of the fifteen PCV15 Ps standards together with the target Ps serotype missing (knockout).

### Preparation of PCV vaccine sample for free Ps analysis

The CRM197-conjugated Ps species were removed from the vaccine product by an affinity capture step. In short, CRM197 conjugated Ps were removed from the vaccine solution by immunoprecipitation with Pierce™ Protein A/G magnetic beads coated with anti-CRM197 monoclonal antibodies (mAbs) at 20 µg/mL each. After removal of the magnetic beads from the solution, the supernatant contained only unconjugated free Ps and was used to bind each anti-ST mAb in the antibody-binding reactions and analyzed on SEC.

### Preparation of anti-ST mAb binding reactions for free Ps analysis

The PCV Ps standards were bound to the corresponding anti-ST mAb in the same or similar fashion that described in Supplementary Table 9a (Standard Binding Table) to generate a serotype-specific standard curve for that type. The reactions are incubated at ambient temperature for one or two hours before injected on HPLC. Each Ps serotype has its own standard curve.

The vaccine solution prepared from immunoprecipitation with anti-CRM197 mAbs was used to bind each anti-ST mAb as in Supplementary Table 9b. Each vaccine preparation will bind to all 15 anti-ST mAbs individually. The reactions are incubated at ambient temperature for the same time period as the Ps standards, before injected on HPLC.

### Multiplex binding reactions for PCV total Ps (conjugated Ps + free Ps) analysis

A mixed PCV standard that contains 1 µg/mL each of ST4, ST5, ST6A, and ST14 total Ps (conjugated Ps + free Ps) was provided by our vaccine formulation group. The PCV24 vaccine^53^ was also provided by our vaccine formulation group.

The PCV standard was multiplexed with the FLR-labeled mAb cocktail (FLR-mAbs) as in Supplementary Table 10 and incubated at ambient temperature for two hours before HPLC analysis. The PCV24 vaccine was multiplexed with FLR-mAbs in the same fashion as in Supplementary Table 11 and incubated for the same time period as the standards before analysis.

### Size-exclusion chromatography (SEC) conditions

The chromatography system was set either on a Waters Alliance (Waters Corporation, Milford, MA), or an Agilent 1260 Infinity II Bioinert system (Agilent, DE, USA), equipped with a quaternary pump, sample manager, column compartment, fluorescence (FLR) and photodiode array (PDA or DAD) detectors. The FLR was detected using excitation (*Ex*) wavelength at 280 nm and emission (*Em*) wavelength at 352 nm (*Ex/Em* 280 nm/352 nm). UV absorbance was recorded at 280 nm (A280).

For detection involving FLR-labeled mAbs, the FLR detections were set at Ex/Em wavelengths corresponding to the FLR tag on the mAbs. Four FLR detection channels were set on the Waters Alliance HPLC system in a single method to allow simultaneous detections of signal carried from four labeled mAbs. The anti-ST4-AF350 was detected at *Ex/Em* of 346 nm/442 nm, anti-ST5-AF430 at 433 nm/541 nm, anti-ST6A-AF555 at 555 nm/565 nm, anti-ST14-AF633 at 633 nm/647 nm.

General chromatography conditions are listed in Supplementary Table 12 (Chromatography Condition Table). Either Chromatography Condition-A or Chromatography Condition-B was used for a certain serotype.

### SEC-MALS-UV-RI analysis of antibody–polysaccharide complex (APC)

The SEC-MALS-UV-RI analysis was performed in the method and chromatography system below. The chromatography condition was the same to the Chromatography condition-A in Supplementary Table 12, except a Tosoh TSKgel G4000PWxL column (7.8 × 300 mm) was used. A multi-angle light scattering (MALS) detector (MiniDAWN^®^) and a refractive index (RI) detector (Optilab^®^ T-rEX) (Wyatt Technology Corp., Santa Barbara) were added to the system and connected in tandem with the UV detector. All data were collected and analyzed with ASTRA 7 software. A first-order fit Zimm formalism with Astra protein conjugate method was used for Mw calculations^47–49^.

Anti-ST1 mAb was complexed with ST1 polysaccharide (Ps) standard at different mAb/Ps ratio as in Supplementary Table 13. The binding reactions were incubated at ambient temperature for 2 h and injected on the SEC-MALS-UV-RI system for analysis.

### Reporting summary

Further information on research design is available in the Nature Research Reporting Summary linked to this article.

## Supplementary information

Supplementary information

REPORTING SUMMARY

## Data Availability

The datasets generated and/or analyzed during the current study are available from the corresponding author upon reasonable request.
