# Supplementary material for: Antibody enhanced HPLC for serotype-specific quantitation of polysaccharides in pneumococcal conjugate vaccine
Source: NPJ Vaccines. 2023 Jan 23;8:2. doi: 10.1038/s41541-022-00584-9 (PMC9869843; doi:10.1038/s41541-022-00584-9)
Supplement: Supplementary file 1 — Supplementary information [file 41541_2022_584_MOESM1_ESM.pdf]

# Supplementary information

**Supplementary Figure 1. Example of pneumococcal Ps repeating unit structure (ST4)**

[→3)-β-D-Man*p*NAc-(1→3)-α-L-Fuc*p*NAc-(1→3)-α-D-Gal*p*NAc-(1→4)-α-D-Gal*p*-2,3(*S*)Pyr(1→]$_n$

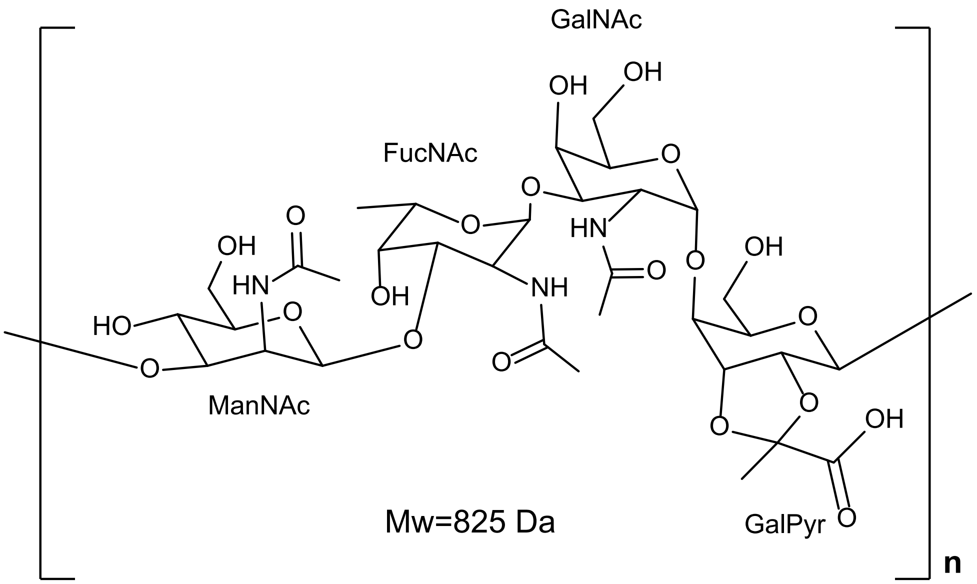


**Supplementary Table 1. Antibody polysaccharide binding reactions for ST4 Ps standard. The first 5 Ps standards (STD1-5) demonstrated linear response to Ps concentration.**

| Binding reaction | 1 µg/mL ST4 Ps (µL) | 0.1 mg/mL anti-ST4 mAb (µL) | Binding Buffer (µL) | Total Vol (µL) | mAb/Ps (µg/µg) | [Ps] (µg/mL) | APC FLR peak area | STD1-5 $R^2$ |
|---|---|---|---|---|---|---|---|---|
| STD1 | 2 | 20 | 178 | 200 | 1000 | 0.01 | 40 | |
| STD2 | 5 | 20 | 175 | 200 | 400 | 0.025 | 98 | |
| STD3 | 10 | 20 | 170 | 200 | 200 | 0.05 | 212 | 0.9998 |
| STD4 | 20 | 20 | 160 | 200 | 100 | 0.1 | 429 | |
| STD5 | 30 | 20 | 150 | 200 | 67 | 0.15 | 655 | |
| STD6 | 50 | 20 | 130 | 200 | 40 | 0.25 | 930 | |
| STD7 | 75 | 20 | 105 | 200 | 27 | 0.375 | 1076 | |

**Supplementary Figure 2. Vaccine ST4 free polysaccharide concentration measured with at different binding reaction time points**

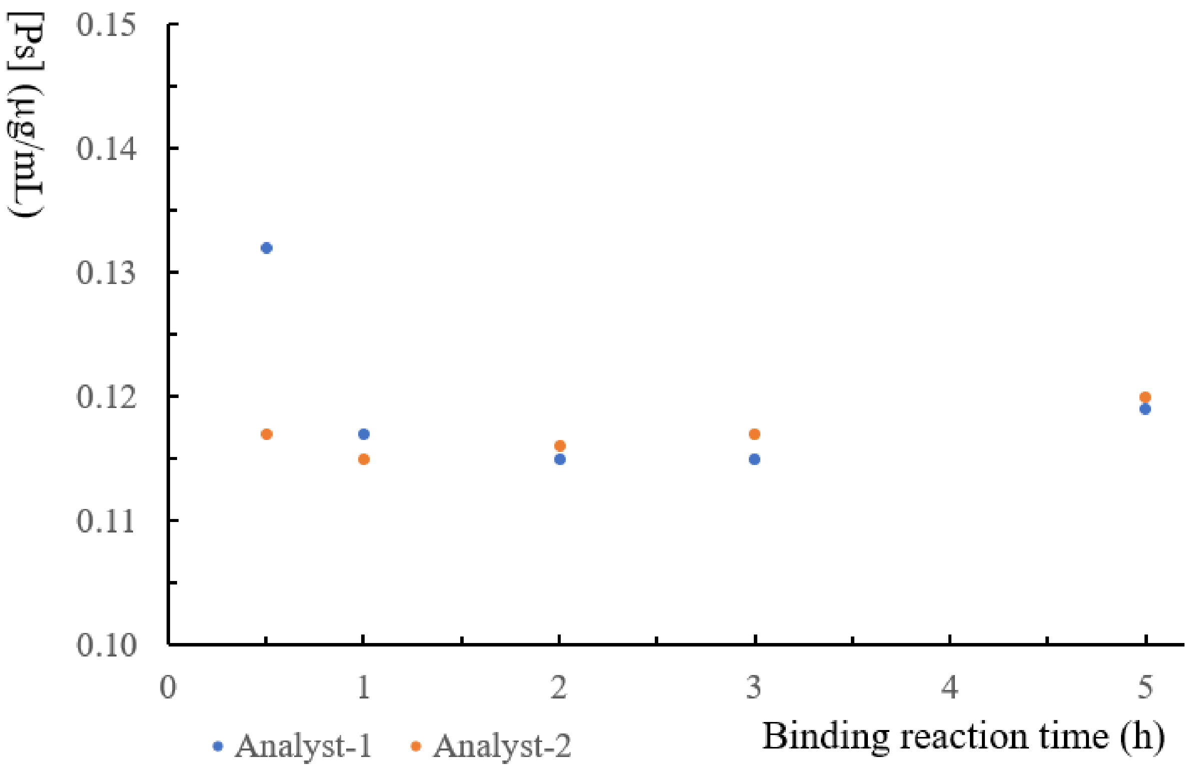


**Supplementary Table 2. Vaccine ST4 free polysaccharide concentration measured with at different binding reaction time points**

| PCV15 | Analyst-1/Instrument-1 | | | | Analyst-2/Instrument-2 | | | |
|---|---|---|---|---|---|---|---|---|
| Binding time (h) | RSQ | PCV15 [ST4] (µg/mL) | Average (1-5h) | %CV (1-5h) | RSQ | PCV15 [ST4] (µg/mL) | Average (1-5h) | %CV (1-5h) |
| 0.5 | 0.9980 | 0.132 | Excluded | | 0.9999 | 0.117 | Excluded | |
| **1** | 0.9999 | 0.117 | 0.117 | 1.6 | 0.9998 | 0.115 | 0.117 | 1.8 |
| **2** | 0.9999 | 0.115 | | | 0.9998 | 0.116 | | |
| **3** | 0.9998 | 0.115 | | | 0.9998 | 0.117 | | |
| **5** | 0.9995 | 0.119 | | | 1 | 0.12 | | |
| Average [ST4] (µg/mL) (2 analysts) | | 0.117 | | | | | | |
| %CV (2 analysts) | | 1.6 | | | | | | |

**Supplementary Figure 3. Compare anti-6A mAb binding reaction with ST6A Ps (green trace) with binding reaction with ST6A knockout (KO) sample (blue trace) on SEC chromatography. Antibody polysaccharide complex (APC) was only observed on the binding reaction with ST6A Ps. Specificity was observed for all PCV15 anti-ST mAbs.**

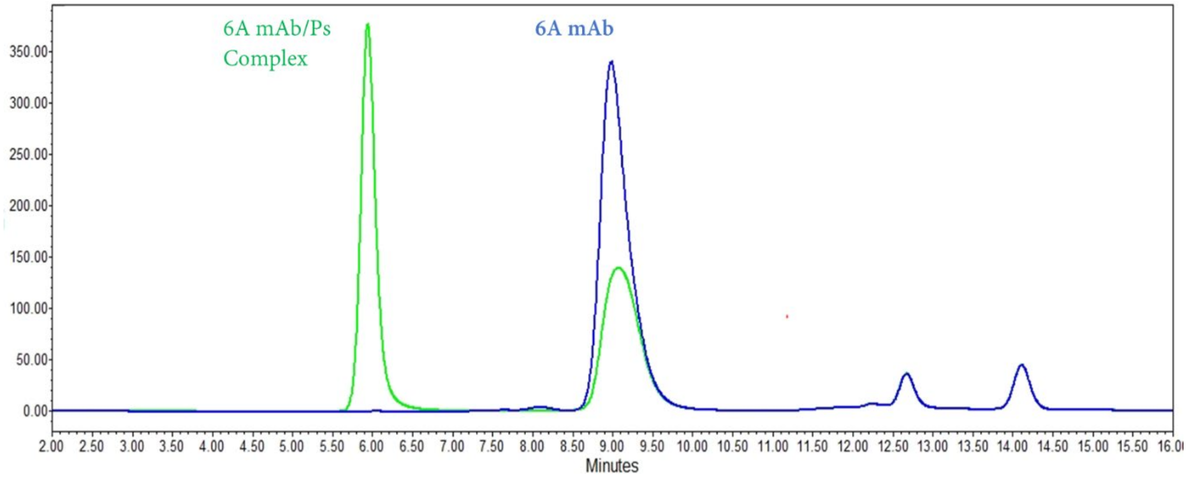


**Supplementary Figure 4. Examples of assay standard curves**

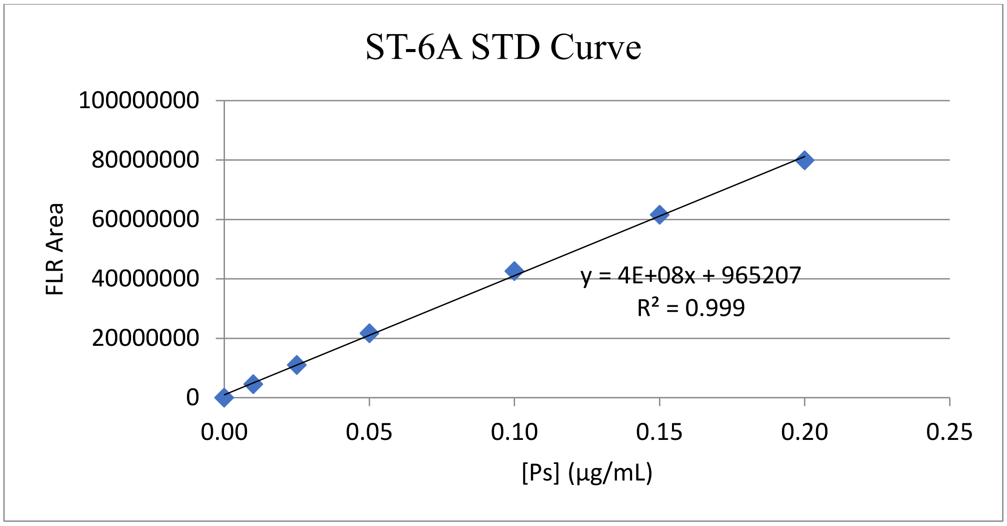

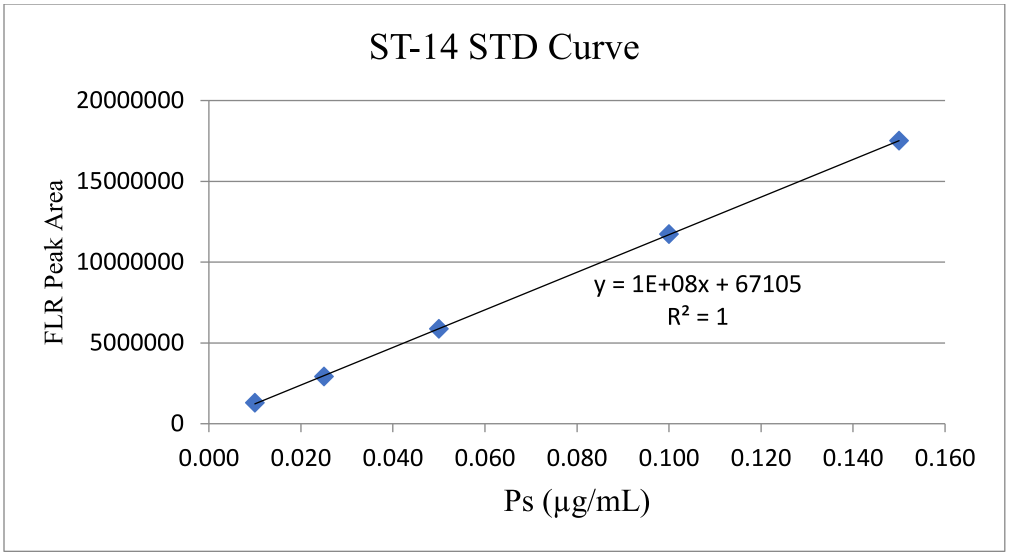


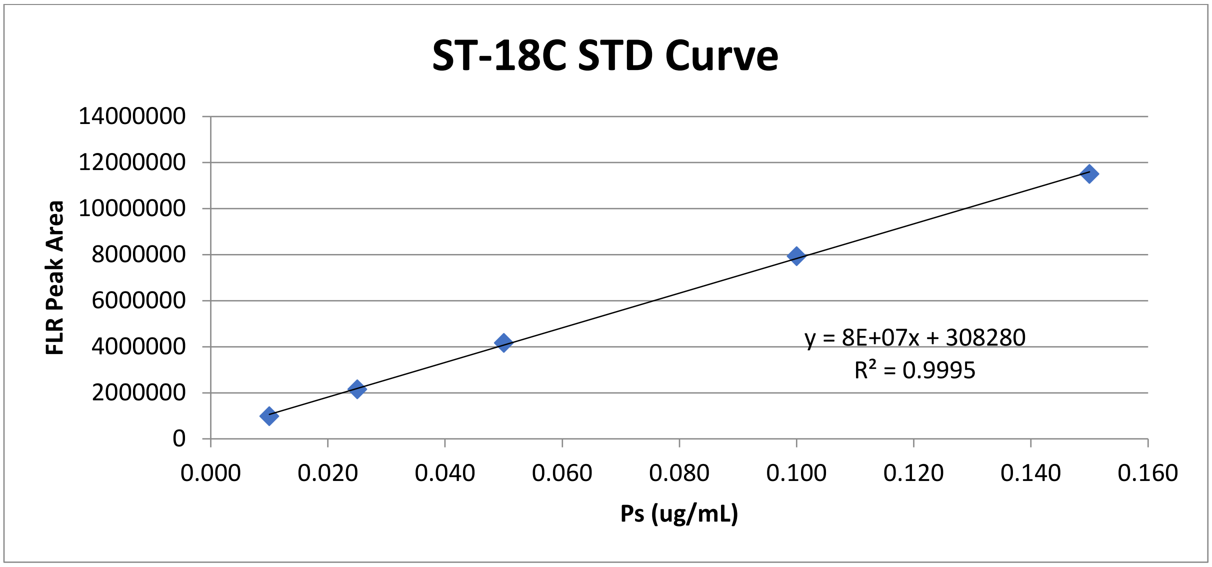


**Supplementary Figure 5. Comparison of anti-ST mAb binding to Ps standards with binding to vaccine free Ps.**

**5-1a. Anti-ST14 mAb binding to ST14 standards**

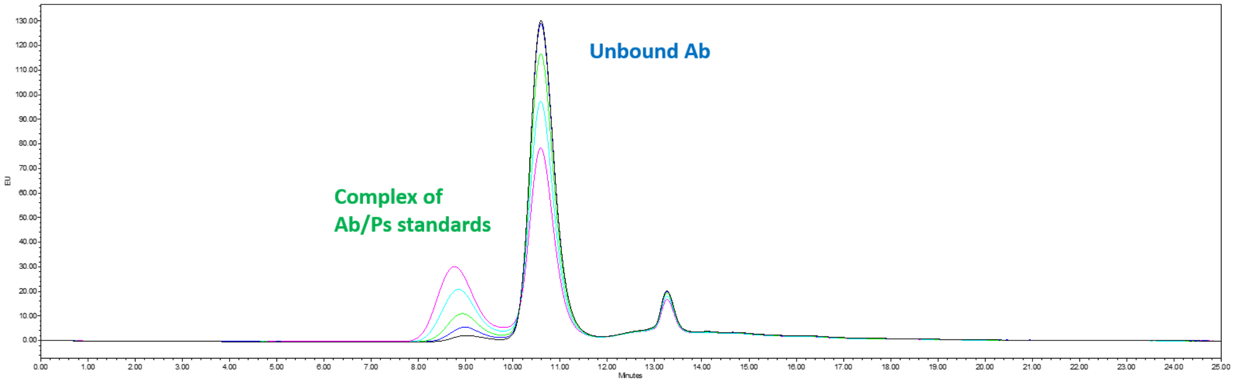


5-

**5-1b. Anti-ST14 mAb binding to PCV**

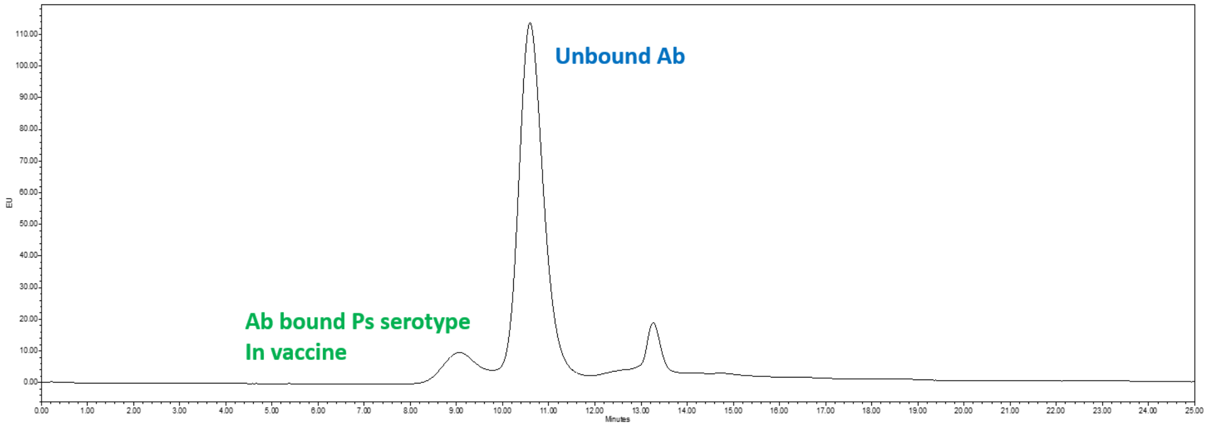


**5-2a. Anti-7F mAb binding to ST7F standards**

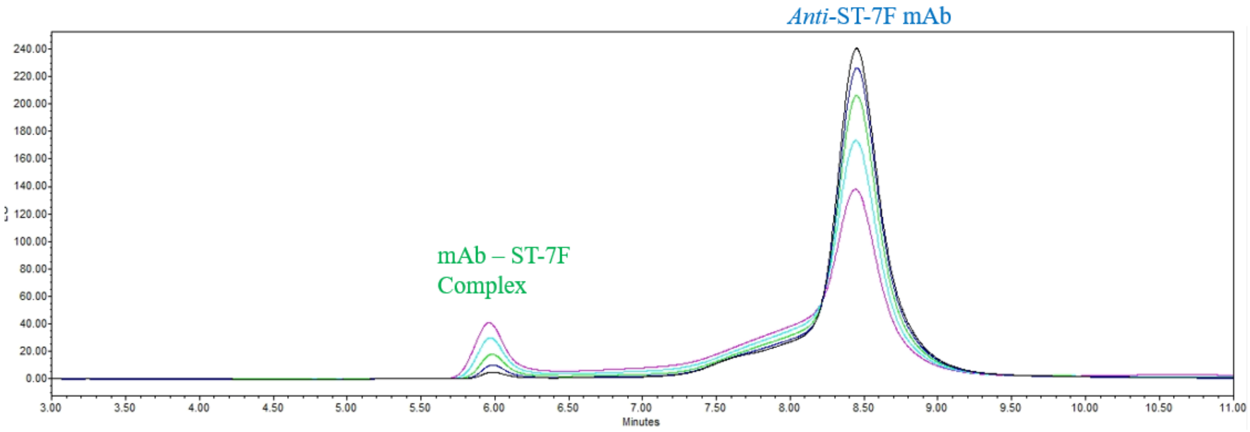


**5-2b. Anti-7F mAb binding to PCV15**

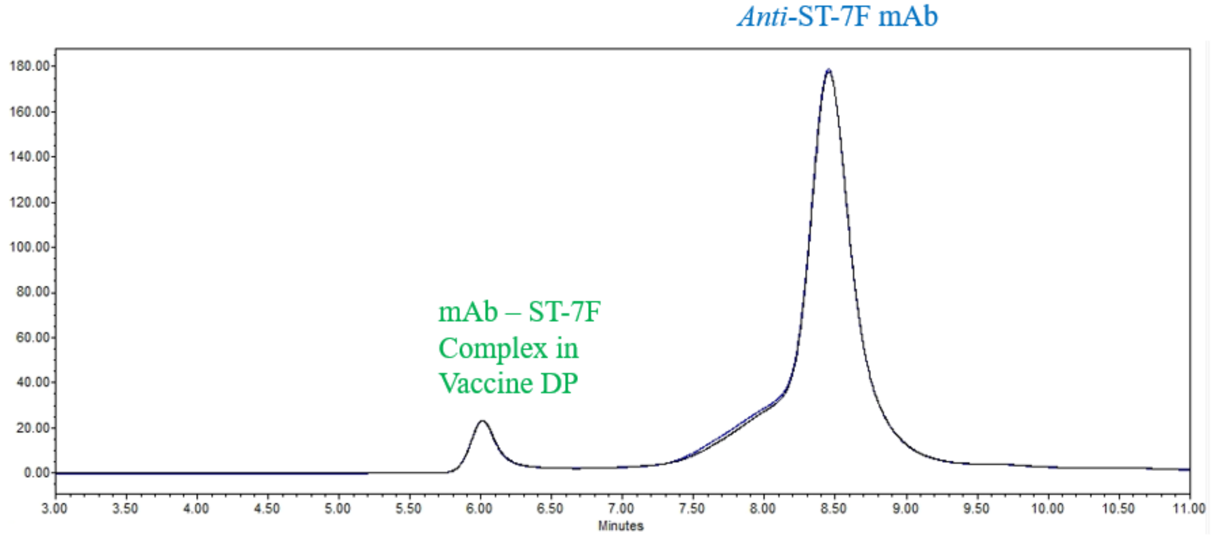


**Supplementary Table 3. Spike recovery of free Ps for six representative PCV15 serotypes**

| PCV15 Serotype | ST4 | ST5 | ST6A | ST9V | ST14 | ST19A |
|---|---|---|---|---|---|---|
| STD curve $R^2$ | 0.9998 | 0.9999 | 0.9999 | 0.9994 | 0.9997 | 0.9999 |
| STD curve intercept | -47562.14571 | -76257.27711 | 853659.4819 | -24030.67809 | -257467 | -305060 |
| STD curve slope | 272223467.8 | 106410195.2 | 581812530.1 | 181277138.5 | 156256712 | 215599880 |
| Spike control FLR | 6458648 | 2536243 | 14115115 | 3719461 | 2740630 | 8312623 |
| LLOQ Spike FLR | 9861719 | 4002479 | 22449281 | 6960319 | 5447363 | 11594188 |
| Nominal Spike FLR | 26282875 | 10561033 | 56552676 | 17525494 | 14864703 | 24152543 |
| Spike control [Ps] (µg/mL) | 0.0239 | 0.0246 | 0.0228 | 0.0207 | 0.0192 | 0.0400 |
| LLOQ Spike [Ps] (µg/mL) | 0.0364 | 0.0383 | 0.0371 | 0.0385 | 0.0365 | 0.0552 |
| Nominal Spike [Ps] (µg/mL) | 0.0967 | 0.1000 | 0.0957 | 0.0968 | 0.0968 | 0.1134 |
| LLOQ Spike Recovered [Ps] (µg/mL) | 0.0125 | 0.0138 | 0.0143 | 0.0179 | 0.0173 | 0.0152 |
| Nominal Spike Recovered [Ps] (µg/mL) | 0.0728 | 0.0754 | 0.0729 | 0.0762 | 0.0776 | 0.0735 |
| LLOQ Spike (µg/mL) | 0.015 | 0.015 | 0.015 | 0.015 | 0.015 | 0.015 |
| Nominal Spike (µg/mL) | 0.075 | 0.075 | 0.075 | 0.075 | 0.075 | 0.075 |
| % Spike Recovery at LLOQ | **83** | **92** | **95** | **119** | **115** | **101** |
| %Spike Recovery at Nominal | **97** | **101** | **97** | **102** | **103** | **98** |

**Supplementary Table 4. Free Ps concentrations from antibody assisted HPLC, sandwich ELISA and expected from standard formulation.**

| PCV15 Serotype | 1 | 3 | 4 | 5 | 6A | 14 | 19A |
|---|---|---|---|---|---|---|---|
| Free [Ps] from Ab-HPLC (µg/mL) | 0.33 | 0.39 | 0.11 | 0.43 | 0.13 | 0.14 | 0.76 |

| | | | | | | | |
|---|---|---|---|---|---|---|---|
| Free [Ps] expected from standard formulation (μg/mL)[a] | 0.3 | 0.3 | 0.2 | 0.3 | 0.1 | 0.1 | 0.5 |
| Free [Ps] from ELISA on a similar batch (μg/mL)[b] | 0.29 | 0.32 | 0.32 | 0.38 | 0.08 | 0.08 | 0.71 |

a: Free [Ps] expected from standard formulation was calculated from theoretical free Ps input for a standard formulation batch.
b: Free [Ps] from ELISA was obtained from measurement of a similar PCV15 batch by sandwich ELISA. Sandwich ELISA was performed following procedure below.

All antibodies were in-house generated serotype specific monoclonal antibodies used at optimized concentrations in the range between 1-3 μg/mL. Plates were washed 3X with wash buffer between each step (Tris-buffered saline + 0.05% polysorbate-20). Maxisorp high-binding 96-well plates (Nunc) were coated (100 μL/well) with serotype specific capture monoclonal antibodies (Merck & Co., Inc., Rahway, NJ, USA) in 1M $(NH_4)_2SO_4$ coating buffer. Following a 1 hour incubation with shaking, plates were blocked for 15 min with assay buffer comprised of 1% (w/v) BSA, 10 mM Tris, 0.15 NaCl, and 0.05 % (v/v) Ps-20, pH 8. References were applied as a serially diluted 11-point curve and samples were plated in 3 replicates (each replicate consisting of 4 pseudo-replicate wells). Samples and references were incubated for 1h at room temperature with shaking after which 100 μL/ well of detection solution was added. The detection solution consisted of serotype specific detection antibodies and an alkaline-phosphatase-labeled anti-species secondary antibody (Jackson ImmunoResearch, cat #111-055-046). Binding was visualized by incubating plates for 35 min with 4-methylumbelliferyl phosphate substrate (Virolabs, XPHOS-100) and reading the resulting fluorescent signal with a Spectramax GeminiEM plate reader (Molecular Devices, CA) at 360 nm excitation and 440 nm emission.

**Supplementary Figure 6. Alexa Fluor™ labeled anti-ST mAbs for multiplex HPLC assay**

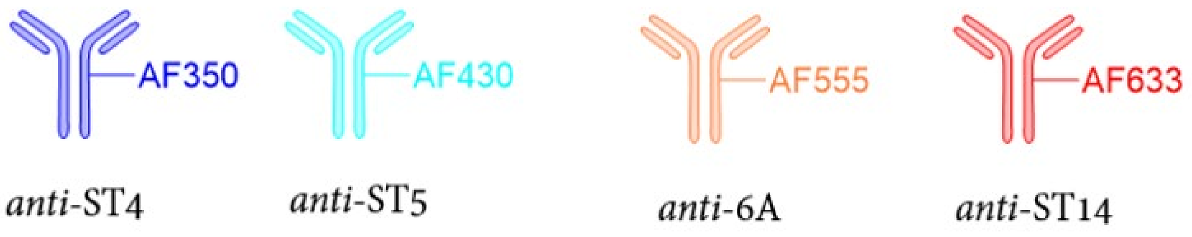


**Supplementary Figure 7. Detection of Alexa Fluor™ 555 labeled anti-6A mAb (anti- ST6A-AF555) on four FLR channels (Ex/Em of 433nm/541nm, 555nm/565nm and 633nm/647nm).**

Anti- ST6A-AF555 can only be detected on 555nm/565nm, not on any other three channels. The channel-specificity was also observed for all other three FLR labeled mAbs (anti-ST4-AF350 only detected at Ex/Em of 346nm/442nm, anti-ST5-AF430 at 433nm/541nm, anti-ST14-AF633 at 633nm/647nm).

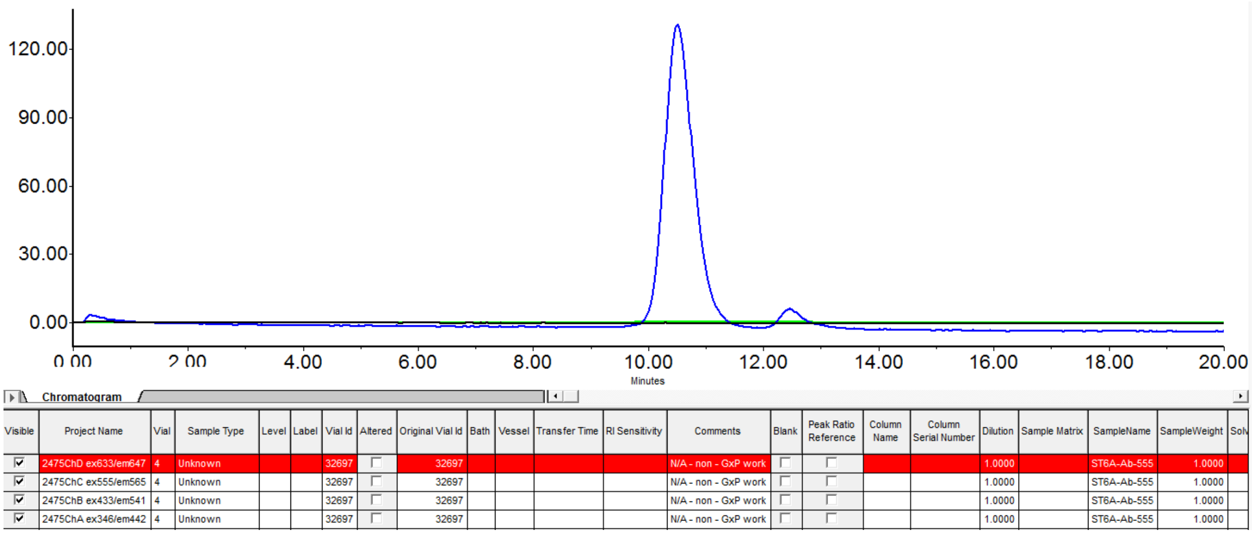


| Visible | Project Name | Vial | Sample Type | Level | Label | Vial Id | Altered | Original Vial Id | Bath | Vessel | Transfer Time | RI Sensitivity | Comments | Blank | Peak Ratio Reference | Column Name | Column Serial Number | Dilution | Sample Matrix | SampleName | SampleWeight | Solv |
|---|---|---|---|---|---|---|---|---|---|---|---|---|---|---|---|---|---|---|---|---|---|---|
| ☑ | 2475ChD ex633/em647 | 4 | Unknown | | | 32697 | ☐ | 32697 | | | | | N/A - non - GxP work | ☐ | ☐ | | | 1.0000 | | ST6A-Ab-555 | 1.0000 | |
| ☑ | 2475ChC ex555/em565 | 4 | Unknown | | | 32697 | ☐ | 32697 | | | | | N/A - non - GxP work | ☐ | ☐ | | | 1.0000 | | ST6A-Ab-555 | 1.0000 | |
| ☑ | 2475ChB ex433/em541 | 4 | Unknown | | | 32697 | ☐ | 32697 | | | | | N/A - non - GxP work | ☐ | ☐ | | | 1.0000 | | ST6A-Ab-555 | 1.0000 | |
| ☑ | 2475ChA ex346/em442 | 4 | Unknown | | | 32697 | ☐ | 32697 | | | | | N/A - non - GxP work | ☐ | ☐ | | | 1.0000 | | ST6A-Ab-555 | 1.0000 | |

**Supplementary Table 5. Multiplex standard curve from binding of an anti-ST-AF cocktail with a multi-valent PCV standard that contains ST4, ST4, ST6A and ST14 conjugates**

| STD Curve | ST4 | ST5 | ST6A | ST14 |
|---|---|---|---|---|
| [Ps] (µg/mL) | FLR350 | FLR430 | FLR555 | FLR633 |
| 0.025 | 13862908 | 3271276 | 14629276 | 1191432 |
| 0.05 | 26449246 | 6062365 | 28413513 | 2344125 |
| 0.1 | 48912423 | 11303380 | 54599861 | 4446046 |
| 0.15 | 73535345 | 16928391 | 84152265 | 6757758 |
| 0.2 | 87356619 | 20278356 | 104312971 | 8126979 |
| **RSQ** | **0.991** | **0.993** | **0.997** | **0.993** |
| Intercept | 4951848 | 1137529 | 2465360 | 323634 |
| Slope | 429252002 | 99344993 | 521487784 | 40472705 |

**Supplementary Table 6. Measurement of total Ps concentrations from 4 PCV24 serotypes in a single HPLC run**

| PCV24 total Ps | ST4 | ST5 | ST6A | ST14 |
|---|---|---|---|---|
| STD curve $R^2$ | 0.9911 | 0.9926 | 0.9966 | 0.9928 |
| Intercept | 4951848 | 1137529 | 2465360 | 323634 |
| Slope | 429252002 | 99344993 | 521487784 | 40472705 |
| FLR channel | FLR350 | FLR430 | FLR555 | FLR633 |
| Peak area | 52581520 | 11054715 | 49747133 | 4163950 |

| | | | | |
|---|---|---|---|---|
| Binding Rx [Ps] (µg/mL) | 0.111 | 0.100 | 0.091 | 0.095 |
| Sample dilution | 80 | 80 | 80 | 80 |
| PCV24 total [Ps] (µg/mL) | 8.9 | 8.0 | 7.3 | 7.6 |
| %diff from target | 11.0 | 0.2 | 9.3 | 5.1 |

**Supplementary Table 7. Species and source of *anti* polysaccharide mAbs**

| **Serotype** | **1** | **3** | **4** | **5** | **6A** | **6B** | **7F** | **9V** |
|---|---|---|---|---|---|---|---|---|
| mAb Species | *Ms* | *Hu* | *Hu* | *Hu* | *Hu* | *Hu* | *Hu* | *Hu* |
| mAb Clone Source | UAB (Hyp1G5.1) | In-house | In-house | In-house | In-house | In-house | In-house | In-house |
| **Serotype** | **14** | **18C** | **19A** | **19F** | **22F** | **23F** | **33F** | |
| mAb Species | *Ms* | *Hu* | *Hu* | *Hu* | *Hu* | *Hu* | *Hu* | |
| mAb Clone Source | In-house | In-house | In-house | UAB (Hyp19FG5.1) | In-house | In-house | In-house | |

In-house: from our in-house antibody generation/reagent teams
UAB: The University of Alabama at Birmingham
*Ms: Mouse; Hu: Human*

**Supplementary Table 8. Dilution of *anti* polysaccharide mAbs before binding reactions**

| **Anti Serotype** | **1** | **3** | **4** | **5** | **6A** | **6B** | **7F** | **9V** | **14** | **18C** | **19A** | **19F** | **22F** | **23F** | **33F** |
|---|---|---|---|---|---|---|---|---|---|---|---|---|---|---|---|
| Target [mAb] (mg/mL) | 0.10 | 0.10 | 0.10 | 0.10 | 0.10 | 0.30 | 0.05 | 0.10 | 0.10 | 0.10 | 0.15 | 0.10 | 0.15 | 0.10 | 0.15 |
| Stock [mAb] (mg/mL) | 4.60 | 4.30 | 4.81 | 4.40 | 0.99 | 11.52 | 1.01 | 4.00 | 3.40 | 1.40 | 4.16 | 6.59 | 1.04 | 1.02 | 1.05 |
| Fold of dilution | 46.0 | 43.0 | 48.1 | 44.0 | 9.9 | 38.4 | 20.2 | 40.0 | 34.0 | 14.0 | 27.7 | 65.9 | 6.9 | 10.2 | 7.0 |
| Stock mAb volume (µL) | 5.4 | 5.8 | 3.1 | 5.7 | 25.3 | 6.5 | 24.8 | 6.3 | 7.4 | 21.4 | 12.6 | 7.6 | 43.3 | 29.4 | 85.7 |
| PBS (µL) | 244.6 | 244 | 146.9 | 244.3 | 224.7 | 243.5 | 475 | 243.8 | 242.6 | 278.6 | 337.4 | 492 | 256.7 | 270.6 | 514 |
| Total mAb solution volume (µL) | 250 | 250 | 150 | 250 | 250 | 250 | 500 | 250 | 250 | 300 | 350 | 500 | 300 | 300 | 600 |

**Supplementary Table 9a. Standard Binding Table**

| Complex | 1 μg/mL ST Ps standard (μL) | 0.1 mg/mL anti-ST mAb (μL) | Binding Buffer (μL) | Total Vol (μL) | [Ps] (μg/mL) |
|---|---|---|---|---|---|
| STD-1 | 2 | 20 | 178 | 200 | 0.01 |
| STD-2 | 5 | 20 | 175 | 200 | 0.025 |
| STD-3 | 10 | 20 | 170 | 200 | 0.05 |
| STD-4 | 20 | 20 | 160 | 200 | 0.1 |
| STD-5 | 30 | 20 | 150 | 200 | 0.15 |

**Supplementary Table 9b. Vaccine Binding Table**

| Free Ps from Vaccine | Vaccine solution Vol (μL) | 0.1 mg/mL anti-ST IgG mAb (μL) | Binding buffer (μL) | Total Vol (μL) | Dilution |
|---|---|---|---|---|---|
| Vaccine free Ps | 20 | 20 | 160 | 200 | 10 |

**Supplementary Table 10. Preparation standard curve for multiplex FLR labeled antibody assay**

| PCV reference standard reaction | 1 μg/mL Ref STD (μL) | 0.2 mg/mL ea of FLR-mAbs (μL)* | Binding Buffer (μL) | Total Vol (μL) | [Ps] (μg/mL) |
|---|---|---|---|---|---|
| PCV-REF-1 | 5 | 15 | 180 | 200 | 0.025 |
| PCV-REF-2 | 10 | 15 | 175 | 200 | 0.05 |
| PCV-REF-3 | 20 | 15 | 165 | 200 | 0.1 |
| PCV-REF-4 | 30 | 15 | 155 | 200 | 0.15 |
| PCV-REF-5 | 40 | 15 | 145 | 200 | 0.2 |

**Supplementary Table 11. Preparation PCV24 for multiplex FLR labeled antibody assay**

| PCV24 reaction | PCV24 8x Dilution (μL) | 0.2 mg/mL ea of FLR-mAb (μL)* | Binding Buffer (μL) | Total Vol (μL) | Total dilution |
|---|---|---|---|---|---|
| PCV24-8x dilution[a] | 20 | 15 | 165 | 200 | 80 |

a: PCV24 was diluted 8-fold in binding buffer before added into the binding reaction.

*: FLR-mAb is a cocktail of four FLR labeled mAbs that consists of anti-ST4-AF350, anti-ST5-AF430, anti-ST6A-AF555 and anti-ST14-AF633. Concentration for each mAb is 0.2 mg/mL.

**Supplementary Table 12. Chromatography Condition Table**

| Chromatography conditions | Chromatography condition-A | Chromatography condition-B |
|---|---|---|

| Column | TOSOH TSKGel GMPWxL (7.8 x 300 mm), 10 µm | Shodex PROTEIN KW-803 (8.0 x 300 mm), 5 µm |
|---|---|---|
| Flow Rate (isocratic) (mL/min) | 0.8 | 1.0 |
| Injection Volume (µL) | 80 | |
| Mobile Phase | 10 mM Bis-Tris-HCl, 300 mM NaCl, pH 7 | |
| Column Temp (ºC) | 35 | |
| Sample Tray (ºC) | 6 | |

**Supplementary Table 13. APC binding reactions for SEC-UV-MALS-RI analysis**

| APC Complex Reaction | mAb/Ps (mg/mg) | 1.0 mg/mL Anti-ST1 mAb (µL) | 0.25 mg/mL ST1 Ps (µL) | Total reaction Vol (µL) | HPLC INJ (µL) |
|---|---|---|---|---|---|
| Complex-1 reaction | 0.25 | 2.5 | 40 | 42.5 | 20 |
| Complex-2 reaction | 0.50 | 5.0 | 40 | 45.0 | 20 |
| Complex-3 reaction | 1.00 | 10.0 | 40 | 50.0 | 20 |
